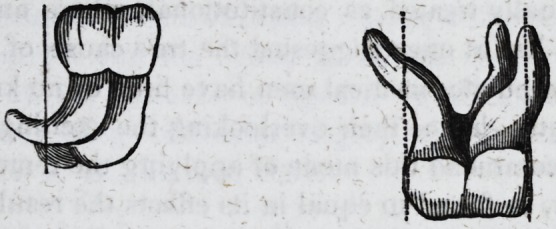# A Much Less Painful and More Scientific Method of Extracting Teeth

**Published:** 1851-04

**Authors:** 


### BIBLIOGRAPHICAL DEPARTMENT.

*A Much Less Painful and More Scientific Method of Extracting Teeth.* By H. Gilbert, M. R. C. S. L., &c. &c. Carroll Minter: London, pp. 67.

Such is the title of this *very* small pamphlet, written, published and distributed by the inventor of the new dental operating chair, a notice of which we gave in a previous number of this Journal. Possibly some of our readers, like ourselves, may expect to find this work a wonder, and its author a prodigy: if so, their expectations will not be realized by a perusal of the essay. In his preface, the author says, (the italics are our own,)

"The operation for extracting teeth, when effected with instruments duly adapted for its ready and safe performance, and by a person fully acquainted with the anatomy and physiology of the parts to be operated on, is unattended with any danger or risk, and with much less pain than is usually supposed. Yet how few *medical men do extract teeth!* Surgeons who would not shrink from performing the most difficult and painful operations, hesitate, and are very unwilling to practice one in reality so simple, and unattended with danger. And why? Because they have no *confidence in the instruments at present in use;* their *knowledge* of physiology and the principles of mechanics teaches them that they are not adapted for the purpose for which they are intended, and they dread the occurrence of one of the following accidents, any one or all of which may happen, without their being able to prevent it, while at the same time its occurrence would assuredly greatly damage their reputation:—breaking the tooth, fracturing the jaw, lacerating and crushing the gums, and frequent slipping of the instrument, requiring repeated readjustment, especially when the key is used. This last named accident is by no means a trifle, on account of the severe pain it causes, and the intensity of the inflammation which may be set up in consequence. To remedy this has been the object of my labors, the result being the invention of the PATENT FULCRUM AND CHAIR, described in the latter part of my little book. This I have used, and still use, with much success; it has also been employed with *equal benefit by those gentlemen who have purchased licenses from me under the patent.* It will be found to obviate most, if not all, the disadvantages and difficulties at present met with in the performance of this operation, whether the key, forceps or elevator be used; at the same time it lessens the pain usually felt during extraction, diminishes the risk of hemorrhage, &c."

Upon what grounds, we ask, does Mr. Gilbert make this sweeping assertion, that the instruments now generally employed for extraction are in their use attended with the awful results he speaks of? If he means that the majority of *his* professional brethren are practically ignorant of the knowledge and existence of the improved instruments used for this purpose, we readily admit the truthfulness of his remarks. If Mr. Gilbert and his medical brethren have been bungling on with grandpapa's sharked jaw forceps and antediluvian keys and punches, with their elevated notions of physiology and the principles of mechanics to guide them in their practice, it does strike us as marvellously strange that *their knowledge* of mechanics should not have helped them to the construction of suitable instruments, and not to avoid operations on account of the inapplicability of those in use—we presume amongst themselves.

Our author further on observes, in reference to the requisite amount of knowledge necessary for the dentist, "All must acknowledge the necessity for an accurate knowledge of the principles of mechanics, as well as of anatomy and physiology, as far, at least, as regards the teeth and their sockets, if they call to mind the difference in the formation of the crowns of the respective teeth, and also in the degree of *divergence* of their fangs, and their direction. The relative strength, too, of the walls or parietes of the sockets must be taken into account." With the preceding observations we perfectly agree: nay, more; in our opinion, to practice the art of dentistry efficiently, one should be acquainted with the collateral branches of medicine, surgery, pathology, mechanics, &c., &c. Yet, even with a knowledge of these sciences, the practitioner will often find cases that will perplex and annoy him in practice.

Mr. Gilbert, to illustrate more forcibly the superiority of his mode of operating over any other, quotes the opinion of several leading authors as to the modus operandi of using the various extracting instruments: one, in particular, he endeavors, by a strange physiological error on his part, to pervert the proper meaning of; we will, however, give the quotation in question. Robinson, in his work when giving directions for the use of the key instrument, says, "for dilating the corresponding portion of the socket, which, by its lateral enlargement," &c. Mr. Gilbert, in his commentary upon this passage, remarks: "If this passage be fairly translated, instead of reading 'dilating the corresponding portion of the socket, which, by its lateral enlargement,'—we should say, *fracturing* the walls of the socket, which, by its *giving way*—as it is utterly impossible for *bone to dilate*, even under the amount of force applied during the use of the key instrument." Surely our perpendicular author must have forgotten the major portion of his physiology of bone, when he wrote these remarks, for if we remember correctly, and daily experience during our pupilage, both upon a dried skull and a recently dead subject, furnished convincing proofs that bone *will dilate*, where force is applied, even in the latter; whilst in the former, either the crown or fangs of a tooth are broken in any attempt, (even perpendicular,) to remove it from its socket. Moreover, how is it, we ask, that the upper molars are removed, if the socket do not dilate? do not the apices of their roots extend over a larger surface than the neck, which is embraced by the alveolus? if so, in every such case, according to Mr. Gilbert's doctrine, either the alveolus is fractured or the roots broken off. Every dental practitioner's daily experience upon the living subject will furnish convincing proofs that Mr. Gilbert's physiological knowledge must be extremely limited, when he ventured to propagate such an absurd doctrine, that *living bone will not yield to pressure without fracturing*.

A friend of the writer of this notice has seen Mr. Gilbert operate, and feels convinced it offers no improvement to our already known appliances for the extraction of teeth; and with this disadvantage—it requires more time in the adjustment of the fulcrum upon which the instrument rests in each particular case; and when properly arranged, and the operation commenced, the remaining teeth in the jaw are forcibly drawn in contact with the fulcrum, which must unquestionably cause serious mechanical injury to the teeth and alveolar periosteum. Independently of these disadvantages, the mere sight of the chair and fulcrum is sufficient to deter nine hundred and ninety-nine patients out of a thousand, from availing themselves of its *peculiar* advantages.

The cases cited by Mr. Gilbert to prove its superiority over the usual method, we think, are mere clap-traps, and do not in the least establish a practical fact—for the most of them had been under the hands of surgeons and hospital pupils, and the one Mr. G. considers his trump card, viz. *Lieut. Cotton's* case, several days had elapsed after the first attempt, and here we may suppose that suppuration had done much in raising the tooth from its socket, which enabled Mr. Gilbert to remove it so dexterously.

After setting forth the peculiar advantages of the fulcrum over the common method of operating, the patentee says: "The teeth are extracted *perpendicularly*, or in the line of their axis." We do not clearly understand Mr. Gilbert's sayings and doings, for our friend personally saw Mr. G. operate on a lower molar of quite the usual character, after the right adjustment of the external fulcrum, and he observed that *lateral before perpendicular* motion was given, quite as much as is usual when the forceps are used in the ordinary manner. This Mr. Gilbert said was necessary to *detach* the tooth *from its periosteum*. This is what we should say is the ordinary method of abducting a tooth, which requires no inordinary machinery in the shape of fulcrums to accomplish. And as the patentee positively asserts that all his extractions are done by the *perpendicular action*, we feel curious to know how he would meet the two following cases—

a dens sapientiæ and an upper molar—according to his *fixed* principle of operating: the straight lines in the diagram indicate the perpendicular action. It may be said we have purposely selected isolated cases: not so; any dental practitioner, of any practice or experience in his profession, will bear us out in our remarks, that similar cases to the above are frequently occurring; or, if further proof be wanting, let the dental apparatus of a prepared human skull be examined, and we much question whether Mr. G's theory of perpendicular extraction will not be found to be a mere flash in the pan, as regards its practical utility in the extraction of teeth.

We moreover advise Mr. Gilbert, when next he goes to press, to rewrite the quotation which has reference to perpendicular extraction, for the sake of consistency, and a due regard to the respectability of the profession of which he is ambitious to be considered a member.

In concluding these observations, we think Mr. G. has manifested considerable ingenuity in a *theoretical* point of view; but, as we before observed, for real practical utility to the dental profession, we consider his invention worse than useless, and we are borne out in these remarks, by the invention itself being laid aside by those who were induced by the novelty to purchase the right of patent for certain towns—a report of which we will give on some future occasion. R.

## Figures and Tables

**Figure f1:**